# The Milan system for reporting salivary gland cytopathology and cyto-histological correlation with special emphasis on cystic lesions—4 years study in a tertiary care institute

**DOI:** 10.3332/ecancer.2025.1870

**Published:** 2025-03-11

**Authors:** Prateek Das, Nibedita Sahoo, Pranita Mohanty, Pallak Batalia, Ankita Pal

**Affiliations:** 1 Department of Oncopathology (Hematopathology), Tata Memorial Centre (HBCH &MPMMCC), Varanasi 221002, UP, India; 2 Homi Bhabha National Institute, Mumbai 400094, India; 3 Department of Pathology, IMS and SUM Hospital, Bhubaneswar 751003, India; 4 Department of Oncopathology, Tata Memorial Centre Sangrur, Sangrur 148001, India; 5 Department of Pathology, Chirayu Medical College and Hospital, Bhopal 462030, MP, India

**Keywords:** Milan system, cytology, cyst, risk of malignancy

## Abstract

**Introduction:**

The Milan System for Reporting Salivary Gland Cytopathology (MSRSGC) was proposed by the American Society of Cytopathology and the International Academy of Cytology to bring uniformity in the reporting and treatment protocol. Cystic component is a common finding in non-neoplastic, benign and malignant lesions of the salivary gland. Fine needle aspiration is challenging in these cases due to pre-analytic error resulting in a high probability of false negativity.

**Aim:**

To classify salivary gland lesions according to MSRSGC with special emphasis on cystic lesions and histological correlation.

**Materials and methods:**

The study included 214 cases of salivary gland Fine needle aspiration cytology (FNACs) and classified according to MSRSGC with calculation of risk of malignancy (ROM) for each category.

**Results:**

The most common age group affected was in the fourth - fifth decade (27.57%) and the common site was parotid gland. The majority of the cases belonged to the Milan category IVA (40.65%) and the least number in categories III and V (4.2%). Histopathology follow up was available in 50% of cases with the maximum number of surgery in category VI (78.5%). The highest ROM was for category VI (90.9%) followed by category V (83.3%). Cystic lesions constituted 22.64%, of which histopathological follow up was available in 64.58% of cases with a high ROM for category III and V(100%).

**Conclusion:**

The ROM for cystic lesions is quite high for all categories as compared to the proposed MSRSGC. We suggest that repeat guided FNAC with a rapid on-site evaluation or ancillary diagnostic techniques can increase the diagnostic accuracy, especially for category I, III and cystic lesions.

## Introduction

Fine needle aspiration cytology (FNAC) is a safe and reliable method for the evaluation of any mass lesion and guides clinicians in proper management. Salivary gland tumours (SGTs) are rare and account for less than 2%–6% of head and neck tumours [1, 2]. According to the GLOBOCAN 2020 report, salivary gland cancers contribute only 0.3% of all cancers [3]. Moreover, there is limited published literature on SGTs in the Indian population [4]. Due to the complications associated with incisional biopsy like fistula formation, tumor implantation and facial nerve damage, FNAC plays an important role in the initial evaluation, diagnosis and subsequent management of salivary gland lesions. The cytomorphological heterogeneity, complexity and overlapping features of benign and low-grade malignancies of salivary gland lesions make it even more challenging to exact categorisation of the tumours, especially differentiating benign lesions from low grade tumours [5]. The accuracy of cytology for categorising and diagnosing various neoplasms is different in various studies and ranges from 48% to 94% [6]. The Milan System for Reporting Salivary Gland Cytopathology (MSRSGC) categorised salivary gland lesions into six diagnostic categories, along with predicting the risk of malignancy (ROM) and recommendation of clinical management strategies [7–8]. Cystic component is a common finding in non-neoplastic (NN), benign and malignant lesions of the salivary gland. FNAC is challenging in these cases due to pre-analytic error resulting in a high probability of false negativity [9]. Limited articles have studied cystic lesions with reference to the Milan system [10]. The present study was carried out to classify salivary gland lesions according to MSRSGC with special emphasis on cystic lesions and histopathological correlation.

## Material and methods

This is a hospital-based retrospective study conducted on all salivary gland swellings over a period of 4 years and the data was retrieved from the hospital database after approval of the Institutional Ethical Committee (IEC) bearing a reference number IEC/IMS. SH/SOA/2023/545. A total of 214 cases of salivary gland FNACs from different sites like parotid, submandibular, submental and oral cavity were included. Wherever available, the detailed clinical and radiological data were retrieved from the hospital information system and records. In our institution, FNAC was performed either by aspiration or non-aspiration technique using a 10 mL disposable syringe and 23/24-gauge needle with prior informed consent. Repeat aspiration or ultrasonography-guided aspiration was done if the aspirate was inadequate for reporting or small, cystic and clinically suspicious of malignancy. The air-dried smears were stained with Diff Quik stain and alcohol (95% alcohol) fixed slides were stained with hematoxylin and eosin (H&E) and Papaniculaou stain. The slides were reviewed by two independent pathologists (NS & PM) and segregated into six diagnostic categories as per MSRSGC: category I: non-diagnostic (ND), category II: NN, category III: atypia of undetermined significance (AUS), category IVA: neoplastic benign (NB), category IV B: salivary gland neoplasm of uncertain malignant potential (SUMP), category V: suspicious for malignancy (SFM) and category VI: malignant (M). The histopathological report was considered as the gold standard.

**Figure 1.**
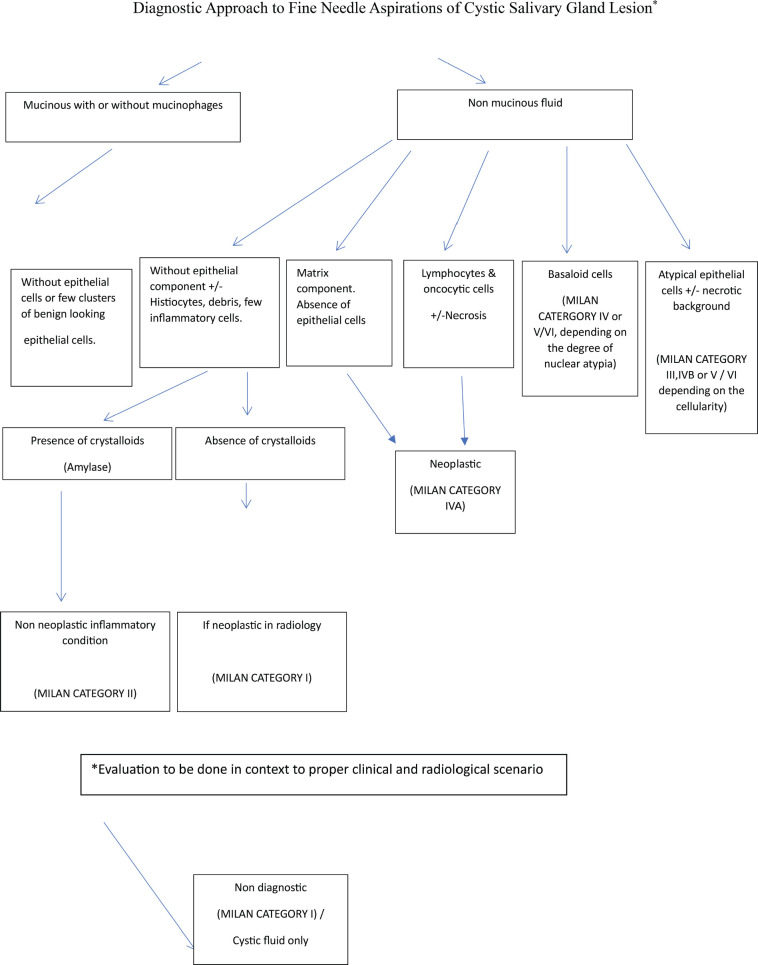
Approach to cystic lesions with prominent cytological features.

Samples were considered as cystic, either by the visually detectable cystic area within the lesion by ultrasonography or when cyst fluid was aspirated during FNAC. The MSRSGC was applied and ROM was calculated for each diagnostic category of cystic lesions. The approach to cystic lesions with prominent cytological features is highlighted in Figure 1.

For statistical calculations, cytological diagnoses were classified as positive (malignant) and negative (benign). Patients with negative cytological diagnosis but later diagnosed as malignant on histopathological examination were considered as false negative, whereas patients with positive cytological diagnosis but later diagnosed benign were taken as false positive. True negative and true positive cases were negative and positive respectively in both cytology and histopathology. Sensitivity, Specificity, positive predictive value (PPV), negative predictive value (NPV) and diagnostic accuracy were calculated. For calculating the risk of neoplasm (RON), the total number of neoplasm (benign and malignant) were taken on a numerator upon total histology follow up cases for the particular category in the denominator and multiplied by 100. Similarly, for ROM, the number of malignant cases were taken in numerator upon the total number of histology follow up cases for each category as denominator, multiplied by 100. The effectiveness of MSRSGC in risk stratifying the salivary gland lesions into benign and malignant was studied by correlating the cytological diagnoses and histopathological outcomes by chi square table and deriving *p* value. A *p* value of <0.05 was considered as significant. For this correlation, the category II and IVa of MSRSGC was clubbed as one group (Benign) and IVB, V and VI were combined into another group (Malignant). Category I and III were not taken into calculation in this.

## Results

A total of 214 cases were included in the study and a male preponderance was observed (124 cases, 57.94%) with a male-to-female ratio of 1.37:1. The most common age group affected belonged to fourth -fifth decade (59 cases, 27.57%) and least was in first decade of life (2 cases, 0.93%). Parotid was the most common site of affection (122 cases, 57.00%) and the lower lip being the least common (1 case, 0.46%). The other sites in decreasing order of frequency are submandibular (77 cases, 35.98%), sublingual and cheek (7 cases, 3.27%), palate (5 cases, 2.33%), and alveolus (2 cases, 0.93%).

In cytology, the majority of cases belonged to the Milan category IVA (87cases, 40.65%) followed by category II (62 cases, 28.97%), category IVB (18 cases, 8.41%), category I (15 cases, 7.00%), category VI (14 cases, 6.54%) and equal number of cases in category III and V (9 cases each, 4.2%). In cytology, the commonest diagnosis in NN category was sialadenosis (15 cases), meanwhile in the NB category it was pleomorphic adenoma (PA) (62 cases) which topped the chart and mucoepidermoid carcinoma (MEC) (9 cases) in the malignant category.

Out of the total 214 cytology cases, 107 cases (50%) were available for histopathological correlation. A maximum number of surgeries were performed in category VI (78.5%) followed by category V, category IVB, category IVA, category I, category III and least number of surgeries were done in category II (24.19%). Among the histopathology diagnosis, the commonest benign tumour was PA (49/107 cases) and malignancy was MEC (16/107). The distribution of cases in cytology according to Milan system, histological correlation with corresponding RON and ROM of each diagnostic category is enlisted in Table 1.

**Figure 2.**
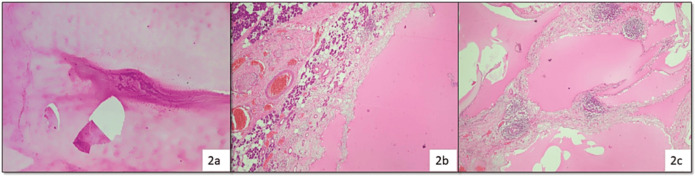
Cytosmear (a): Showing only proteinaceous fluid admixed few lymphocytes and assigned to category I (H&E, 100×) histopathology (b and c): Confirmed the diagnosis of cystic lymphangioma due to the presence of many endothelium lined dilated spaces containing proteinaceous material and lymphoid follicles in the wall (H&E, 100×).

Three cases from ND category turned out to be malignant, i.e., two cases of MEC and a single case of acinic cell carcinoma (ACCA); while benign tumours were two cases each of PA and Warthin’s tumour (WT) and one case of cystic lymphangioma (Figure 2).

**Figure 3.**
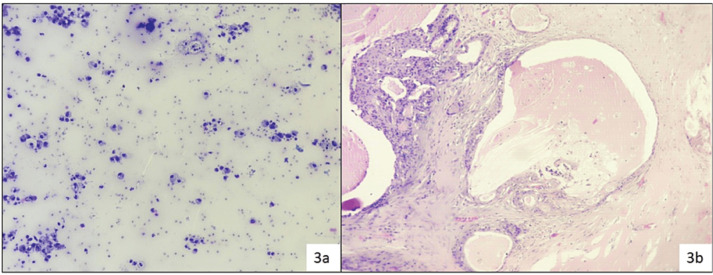
Reported as benign cystic lesion due to the presence of only cyst-macrophages (a): MSRSGC category II (Diff Quik, 200×) histopathology (b): Turned out as LGMEC (H&E, 100×).

In category II (NN), 15 cases (15/62) were evaluated in histopathology, discordant cases were one case of PA reported as Kuttner tumour (chronic sclerosing sialadenitis), one case of low-grade mucoepidermoid carcinoma (LGMEC) reported as benign NN cystic lesion (Figure 3).

**Figure 4.**
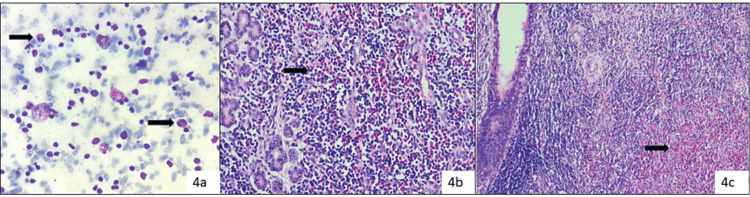
Cat 2: Cytosmears (a): Showed only lymphocytes and reactive lymphoid cells and reported as intra-parotid reactive lymph node (Diff Quik, 400×) (Category II), histopathology (b and c): Confirmed as KD (H&E, 200×) (arrow pointing eosinophils in cytology and histology slides).

In category IVB (SUMP), out of 12 surgeries, 5 cases turned out malignant, i.e., two cases of ADCC as shown in Figure 7 and myoepithelial carcinoma, LGMEC and ACCA (one case each) (Figure 4).

**Figure 5.**
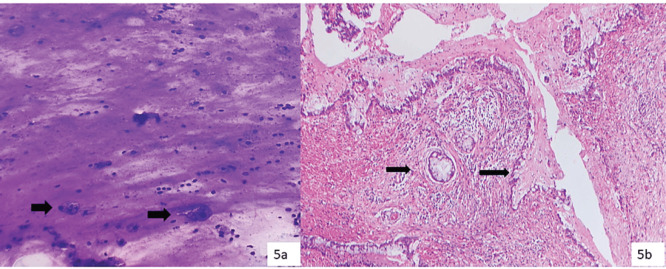
Cytosmears (a): Showing mucinophages (thick arrow) over a mucoid background and assigned category III (Diff Quik, 200×), histopathology (b): Confirmed as LGMEC with many cystic foci lined by mucous cells with mucin vacuoles (thin arrow) (H&E, 200×).

Out of four resected cases of category III (AUS), three cases turned out as MEC and one case as chronic sialadenitis. All the MEC cases had only mucin without or scant diagnostic epithelial components in cytology (Figure 5).

**Figure 6.**
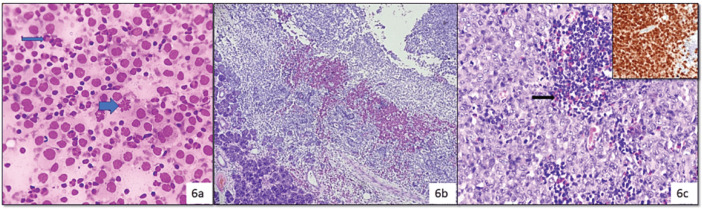
Cytology (a): Showing histiocyte-like cells with moderate pale cytoplasm, round to oval nucleus with grooves (arrow), mitotic figures (thick arrow), admixed lymphocytes and eosinophils (Diff Quik, 400×), histopathology (b): Confirmed as LCS due to presence of nuclear atypia and atypical mitosis (H&E, 100× and 400×), Positive IHC stain for S-100 (Inset in 5A.iii) (Diaminobenzidine, 400×).

In category IVA (NB), out of 48 resected cases the most common diagnosis was PA (35 cases), followed by WT, schwannoma and discordant cases were Langerhans cell sarcoma (LCS) reported as Langerhans cell histiocytosis (LCH) (Figure 6).

**Figure 7.**
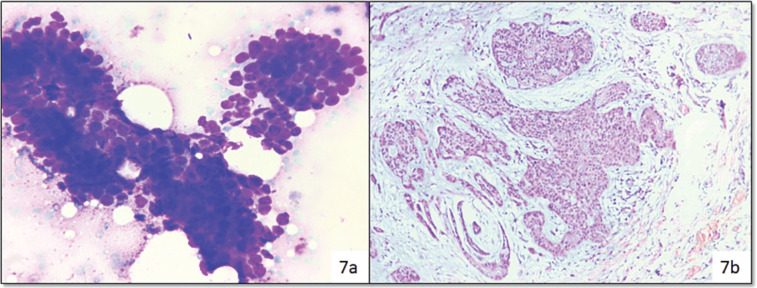
Cytosmear (a): Showing basaloid cells and assigned to category IVb (Diff Quik, 400×), Histopathology (b): Confirmed as ADCC (H&E, 200×).

LGMEC was reported as PA with cystic change, adenoid cystic carcinoma (ADCC) was reported as basal cell adenoma and carcinoma ex pleomorphic adenoma reported as PA. In category IVB (SUMP), out of 12 surgeries, 5 cases turned out malignant, i.e., two cases of ADCC (Figure 7).

**Figure 8.**
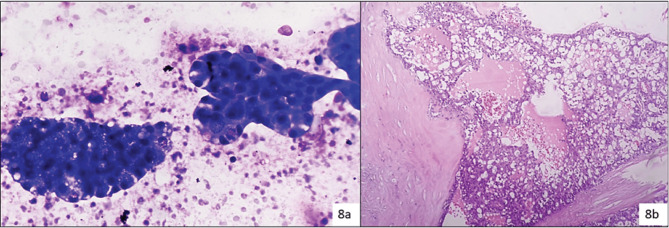
Cytosmear (a): Showing atypical cells with cytoplasmic vacuolation and squamoid appearance, due to low cellularity assigned to category V (possibly MECa) (Diff Quik, 400×), histopathology (b): Confirmed as SCa (H&E, 200×).

One case of low-grade mucoepidermoid carcinoma (LGMEC) was reported as benign NN cystic lesion as shown in Figure 3 and one case of follicular lymphoma was reported as an inflammatory lesion (Figure 8).

**Figure 9.**
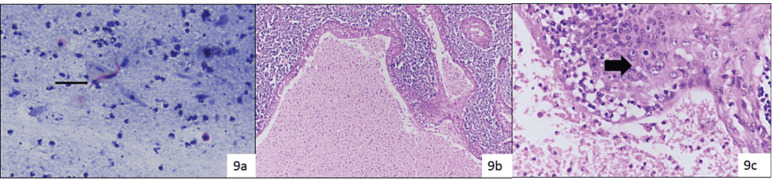
Smears (a): Showing dyskeratotic cells and fibre cells (thin arrow ) over necrotic background suggestive of squamous cell carcinoma and assigned category VI (Papaniculao stain, 400×), histopathology (b and c): Confirmed WT with squamous metaplasia (thick arrow) (H&E, 200× & 400×).

One case as lymphoma (by flowcytometry) and the remaining one case turned out as WT reported as suspicious for MEC. From the Milan category VI (M) (14 cases), 11 cases were resected, out of which a single case was discordant and that turned out to be benign (WT with extensive squamous metaplasia reported as squamous cell carcinoma) while the rest were malignant (Figure 9).

**Figure 10.**
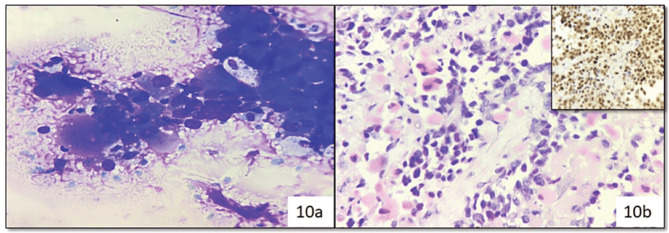
Cytosmear (a): Showing atypical plasmacytoid cells and reported as myoepithelial carcinoma (Category VI) (Diff Quik, 400×), histopathology (b): (H&E, 400×) and immunohistochemistry (Inset: MYO-D1) (Diaminobenzidine, 400×) confirmed metastatic rhabdomyosarcoma.

One case was reported as myoepithelial carcinoma due to plasmacytoid morphology and discretely lying cells with nuclear atypia, which on histopathology and immunohistochemically confirmed as metastatic rhabdomyosarcoma (Figure 10).

Cystic lesions constituted 22.64% of all FNACs (*n* = 48). MSRSGC was applied to all cases and there were 11 (22.9%) ND cases, 12 (25%) NN cases, 3 (6.25%) in AUS category, 8 (16.66%) benign neoplastic, 6 (12.5%) SUMP, 2 (4.16%) SFM and 6 (12.5%) were malignant. Histopathological follow up was available in 64.58% (31/48) cases (Table 2).

The cytology cases were compared with histopathology as the gold standard and the sensitivity, specificity, PPV, NPV, and diagnostic accuracy were calculated as 57.7%, 96.9%, 88.23%, 85.3% and 85.8%, respectively. The overall ROM amongst cystic lesion was 38.46%. The cytological diagnosis and risk stratification by MSRSGC correlated significantly with the histopathological outcome in overall salivary gland lesions (*p* value <0.00001) as well in cyctic lesions (*p* value ≤ 0.004).

## Discussion

FNAC is a well-established procedure for preoperative evaluation of patients with salivary gland lesions. The MSRSGC is currently a user-friendly standard reporting system across institutes for better clinical communication and improved patient care.

We analysed 214 cases in FNAC and follow up biopsy was available in 50% of cases. Histopathological follow up in different studies ranges from 14.9% to 100% [11, 12]. Parotid gland was commonly affected site (57%), followed by the submandibular gland and minor salivary gland, which was comparable with other studies [13]. The present study’s maximum number of cases were in category IV A (NB) followed by category II (NN), which is quite comparable with a study by Karuna *et al* [13]. In the indexed study, the ROM in different categories were ND (37.5%), NN (13.3%), AUS (75%), BN (8.33%), SUMP (41.6%), SFM (83.3%) and malignant category (90.9%), respectively, and is comparable with MSRSGC except for a higher value for category III, which is comparable with studies by Rohilla *et al* [11], Jha *et al* [12] and Archondakis *et al* [14]. The sensitivity and specificity of fine needle aspiration in previous studies range from 54%–98% to 88%–99%, respectively [12,14]. In our study, the sensitivity was 57.7% while specificity was 96.9%. The most common benign neoplasm in our study was PA, while the most common malignant tumour was MEC which is comparable with other studies [13]. The ROM for various diagnostic categories in the previous studies and current studies is depicted in Table 3 [8, 11–12, 14–23].

ND (Category I): This category constituted 7% which is near to the optimum level proposed in MSRSGC [8]. In this category, two types of samples were included, i.e., either non-mucinous cystic fluids or poor quality of specimen, in contest to the clinical scenario. Cystic lesions such as retention cyst, mucocele, lymphoepithelial cyst, WT, mucoepidermoid carcinoma, ACCA, cystic PA or cystadenoma, of low cellularity are usually included in this category [14]. The ROM varies from 6% to 70% in various studies, highlighting the importance of repeat guided FNAC and biopsy for picking up malignancy, especially in institutions with trainee residents like ours.

NN (Category II): In our study, this was the second most common type after benign neoplastic category. We observed two false negative results, one case of MEC (reported as mucus retention cyst) and follicular lymphoma (reported as reactive lymph node). The reason behind false negativity is cellularity and obscuring elements like inflammation or hemorrhage in the background [14]. Prior studies also highlighted false negative diagnosis in cases of lymphoma and were put in NN category as reactive lymph node [22]. One case reported as a reactive intraparotid lymph node turned out to KD. Retrogradely slides reviewed showed along with a polymorphous population of lymphoid cells, admixed eosinophils which were missed. KD is a rare chronic inflammatory disorder of unknown etiology that involves the subcutaneous tissue, lymph nodes or salivary glands of the head and neck region. Albeit, cytology has limited value in KD but should be suspected in cases of reactive lymphoid cells, eosinophils and few Warthin-Finkeldey like giant cells with peripheral blood eosinophilia for proper clinical management [24].

AUS (Category III): We encountered the least number of cases in this category(9/214) with a higher ROM (75%). Aspirate from cystic component with the presence of mucoid material, occasional mucinophages or few intermediate like cells cannot exclude low-grade malignancy like MEC. Rohilla *et al* [11] and Jha *et al* [12] had a higher ROM of 100% in their studies. For this category, MSRSGC recommends repeat USG-guided FNAC and surgery depending on clinico-radiological findings.

Benign neoplastic (Category IVA): A maximum number of cases were in this category (77/214) with a concordance rate in biopsy of 91.66% (44/48 cases), while four cases showed discordant results (Table 1). One interesting case diagnosed as LCS in biopsy was diagnosed as LCH in cytology in a 25-year-old male presented with bilateral submandibular swelling. Retrograde cytology slides reviewed and many mitotic figures including atypical forms were identified in the histiocytic cells LCS is a rare malignant tumour of Langerhans cells with malignant cytological features and an aggressive clinical course. Salivary gland involvement of LCS is extremely rare; however, a possibility should be kept in mind while dealing with histiocytic cells with vesicular chromatin, nuclear grooves having a good number of mitosis over a background containing eosinophils and lymphocytes [25]. Also, in our series, we encountered three cases of schwannoma, which should be differentiated from PA and myoepithelioma of spindle cell morphology. But in schwannoma cells are in tissue fragments and cohesive groups over a myxoid stromal material. One case showed mild nuclear atypia and has been put in the SUMP category. ROM for this group in our study was 8.3% and is comparable with a study by Rohilla *et al* [11] and Archondakis *et al* [14].

SUMP(Category IV B): This category includes cases with features of neoplasm, yet a definite diagnosis cannot be rendered or neoplasm with few atypical features where malignancy cannot be excluded. Cellular benign neoplasms, neoplasms with atypical features, low-grade carcinomas and basaloid neoplasms of salivary gland having morphology resembling benign to low-grade malignancies (like ADCC, epithelial myoepithelial carcinoma or polymorphous adenocarcinoma) were put in this category. Out of 18 (8.4%) cases, 12 cases were classified in histopathology with a ROM of 58.3%. The present study showed a higher side threshold of malignancy risk and is comparable to other studies [5, 14, 17]. One case of PA showed marked atypia of the epithelial component but later turned out as cellular PA. Atypia of the epithelial cells is not uncommon in PAs and the coexistence of poorly differentiated cells will guide diagnosis towards a malignant tumour [26].

SFM( Category V): In this category, the cytological features are in favour of malignancy but a specific diagnosis could not be rendered due to artifacts, limited diagnostic material or admixed benign cellular components. This indeterminate category has a high ROM especially for cystic lesions in our series which is comparable to other studies. One case diagnosed was suspicious of MEC turned out as WT in histopathology. The dirty background from the cystic area probably led to the discordant diagnosis.

Malignant (Category VI): When the cytological findings are diagnostic of malignancy were put in this category. The ROM in our study is comparable with other studies (Table 3). Most diagnostic difficulties occurred in LGMEC and were put in category V, while high-grade tumours with adequate cellularity were easy to categorise [14]. One discordant result in this category was a case of WT with extensive squamous metaplasia reported as metastatic squamous cell carcinoma and this observation was also occurred in other studies [27, 28].

Cystic lesions are challenging with broad differentials and needs radiological correlation for proper categorisation. Major limitations in diagnosis of cystic lesions are the overlapping features of benign and malignant lesions or cystic change of nearby non-salivary gland tissue, like metastatic lymph node or cutaneous cystic lesions. In our study, maximum number of cases were in category II followed by category I which is compatible with a study by Maleki *et al* [29]. In our study, LGMEC was the commonest malignant lesion (78.57%) and is also a common cause of false negative results because of stringy mucin, few macrophages and bland epithelial cells [30]. Literature highlights one-third of cystic salivary gland lesions are neoplastic, in our study the RON was 87.5% which is quite high. The probable explanation is the low sample size and high clinical suspicion leading to surgery. The ROM of different categories is higher for all categories in comparison with MSRSGC is also observed in a study by Maleki *et al* [29]. In the cystic lesions, we observed 100% ROM for category III and V which is 20% and 60% respectively in proposed MSRSGC [8]. The ROM is probable explanation is low cellularity due to cystic lesion leads to false negative results but high clinical suspicion for malignancy leading to surgical intervention.

The limitation of our study is only 50% of cases have follow up data and this may lead to a higher ROM in category III and to some extent in category IV, thus creating an obstacle for proper clinical management. The retrospective nature of our study undermines the credibility of our results, especially cystic fluids which could have been used for cell block studies to increase the diagnostic yield.

## Conclusion

The ROM for cystic lesions is quite high for all diagnostic categories as compared to MSRSGC. We believe repeat guided FNAC for all cases of category III and cystic lesions with a rapid on-site evaluation or ancillary diagnostic techniques can increase diagnostic accuracy. Larger studies, meta-analysis and reviews should be performed for future MSRSGC revisions.

## Conflicts of interest

There is no conflicts of interest.

## Funding

Nil.

## Figures and Tables

**Table 1.** Cyto-histopathological correlation, RON and ROM for each category.

Milan	Cyto diag	No of cases	No of HPE	Benign HPE diagn	Malig HPE dia	Concordant	Discordant	RON	ROM
I (*n* = 15)	ND	15	8 (53.3%)	Lymphangioma(1), PA(2) , WT(2),	MEC(2) ACCA(1)			5 (62.5%)	3 (37.5%)
II (*n* = 62)			15 (24.19%)					4 (26.6%)	2 (13.3%)
	SA	15	0						
	AS/AI	10	0						
	NCL	7	6	NCL(5)	MEC(1)	5	1	1	1
	CS	14	6	PA(2),CS(4)		4	2	2	
	RL	9	2	KD (1)	Follicular lymphoma (1)		2		1
	GL	5							
	Epidermal inclusion cyst	2	1			1			
III (*n* = 9)		9	4 (44.4%)	CS (1)	MEC (3)			3 (75%)	3 (75%)
IVA (*n* = 87)			48 (55.1%)					48 (100%)	4 (8.33%)
	LCH	1	1		LCS		1	1	1
	PA	62	37	PA(35)	LGMEC(1) CXPA (1)	35	2	37	2
	WT	8	3	WT(3)		3		3	
	BST	6	3	Schwannoma (3)		3		3	
	BCA	6	2		ADCC (1)		1	2	1
	Lipoma	1	1	Lipoma		1		1	
	Oncocytic neoplasm	3	1	WT		1	0	1	0
IVB (*n* = 18)			12 (66.675)					12 (100%)	5 (41.6%)
	CPA	8	4	CPA(3)	ADCC (1)	3	1	4	1
	Basaloid neoplasm	6	4	Basal cell adenoma (2)	Epi myo ca (1) ADCC 1	2	2	4	2
	Spindle cell tumor with atypia	2	2	Schwannoma (2)	00	2	0	2	0
	Oncocytic tumor with atypia	2	2	0	LGMEC (1) ACCA (1)	0	2	2	2
V (*n* = 9)			6 (66.6%)					6 (100%)	5 (83.33%)
	Susp of MEC	2	1	WT	-	0	1	1	
	MEC	1	1		SCa	1		1	1
	MEC/SCC	1	1		MEC	1		1	1
	SCC with cystic degn	1							
	LGMEC	1	1		MEC	1		1	1
	susp of lymphoma	1	1		Lymphoma (FLOW)	1		1	1
	Susp of myoepithelial CA	1	1		MEC	1		1	1
VI (*n* = 14)			11 (78.57%)					11 (100%)	10 (90.90%)
	MEC	6	5		MEC(5)	4	1	5	5
	NHL	1	1		DLBCL	1		1	1
	METS SCC	1	1	WT1			1	1	
	ADCC	1	1		ADCC (1)	1		1	1
	High GR CA	2	1		High GR CA			1	1
	SCC	1							
	Myoepithelial CA	2	2		Myoepithelial CA (1) METS RMS (1)	1	1	2	2
Total		212	102			81	14		

ND- Non Diagnostic; PA- Pleomorphic adenoma; WT- Warthin’s tumour; MEC- Mucoepidermoid carcinoma; ACCA- Acinic cell carcinoma; SA- Sialadenosis; AS/AI- Acute Sialadenitis/ Acute inflammatory lesion; NCL-Non neoplastic cystic lesion MEC- Mucoepidermoid carcinoma; CS- Chronic Sialadenitis; RL- reactive lymphadenitis; GL- granulomatous lymphadenitis; LCH- Langerhan cell histiocytosis; PA- Pleomorphic adenoma; LCS- Langerhan cell sarcoma; BST- benign spindle cell tumour; LGMEC- Low grade mucoepidermoid carcinoma; CA EX PA- Carcinoma ex pleomorphic adenoma; BCA- Basal cell adenoma; ADCC- Adenoid cystic carcinoma; CPA- Cellular Pleomorphic adenoma; Epi Myo Ca- Epithelial myoepithelial carcinoma; SCa - Secretory carcinoma; SCC- Squamous cell carcinoma; NHL- Non Hodgkin’s Lymphoma; DLBCL- Diffuse Large B cell Lymphoma; RMS- Rhabdomyosarcoma; METS- Metastatic

**Table 2.** Cyto-histopathological evaluation of cystic lesions (*N* = 48).

Category	No of cases	NO of BX	Histopathology diagnosis	RON	ROM
I	11 (22.9%)	8	Mucus retention cyst(1), Cystic lymphangioma(1), WT(2), PA(2), LGMEC(2)	87.50%	25%
II	12 (25%)	4	Epidermal inclusion cyst, Mucus retention cyst, Lymphoepithelial cyst, LGMEC	25%	25%
III	3 (6.25%)	3	LGMEC(3)	100%	100%
IVA	8 (16.66%)	7	PA(1), Schwannoma(2), WT(4)	100%	00%
IVB	6 (12.5%)	2	Cellular PA, Epithelial myoepithelial carcinoma	100%	50%
V	2 (4.16%)	2	LGMEC	100%	100%
VI	6 (12.5%)	6	WT(1), LGMEC(4), SCa(1)	100%	83.33%
Total	48 (100%)	32(66.6%)		87.5%	43.75%

ROM was highest in category III and V (100%) followed by category VI (83.3%)

**Table 3.** Comparision of category-wise ROM of our study with other studies (%).

	Year of publication	Cat I	Cat II	Cat III	Cat IVA	Cat IVB	Cat V	CatVI
Proposed MSRSGC [8]	2018	25	10	20	<5	35	60	90
Rohilla *et al* [11]	2017	70.4	17.4	100	7.3	50	96	96
Viswanathan *et al* [15]	2018	6.7	7.1	38.9	5	34.2	92.9	92.3
Hollyfield *et al* [16]	2018	38	17	33	4	33	85.7	97.5
Mazzola *et al* [17]	2019	19	11.8	25	5.5	50	71.4	94.6
Wu *et al* [18]	2019	18.3	8.9	37.5	2.9	40.7	100	98.3
Lee *et al* [19]	2019	10	17.5	29.5	0.5	17.1	83.3	100
Jha *et al* [12]	2020	42.86	30.77	100	10.17	0	71.42	100
Archondakis *et al* [14]	2021	33.3	16.7	100	7.7	50	100	91.7
Wang *et al* [21]	2022	11.4	10.9	30.5	2.8	37.7	83.8	97.7
Morand *et al* [22]	2022	21.4	0	50	0	30	100	100
Ratzon *et al* [23]	2023	29	22	37.1	25	62	88	99
Present study	2024	37.5	13.3	75	8.33	41.6	83.3	90.9
